# The Asymptotic Solutions for a Class of Nonlinear Singular Perturbed Differential Systems with Time delays

**DOI:** 10.1155/2014/965376

**Published:** 2014-04-16

**Authors:** Han Xu, Yinlai Jin

**Affiliations:** Science College, Linyi University, Linyi, Shandong 276005, China

## Abstract

We study a kind of vector singular perturbed delay-differential equations. By using the methods of boundary function and fractional steps, we construct the formula of asymptotic expansion and confirm the interior layer at *t* = *σ*. Meanwhile, on the basis of functional analysis skill, the existence of the smooth solution and the uniform validity of the asymptotic expansion are proved.

## 1. Introduction

Singular perturbed differential equations are often used as mathematical models describing processes in biological sciences and physics, such as genetic engineering and the El Nino phenomenon of atmospheric physics [1]. In order to study natural and social processes more accurately, we often construct the models with small delay time and obtain much behavior of corresponding objects. The models are mostly expressed by singular perturbed delay-differential equations. So, singular perturbed delay-differential equations can express the processes more exactly. Studying the singular perturbed delay-differential problem is a very attractive object in the mathematical circle.

In addition, in the study of population models and propagation of epidemic virus, we sometimes require the construction of models. The models are often expressed by singular perturbed delay-differential equations. We can get the equilibrium points of singular perturbed delay-differential equations and confirm the laws of processes. Therefore, using the research methods and theoretical results of singular perturbed delay-differential problems to solve natural and social processes is essential.

In recent years, more and more attention was paid to the study of singular perturbed delay-differential problems [2], especially for scalar boundary value problems [3–5], but vector boundary value problems are rarely seen [6, 7]. Up to now, the vector theory of singular perturbed problems is still not mature. Wang and Ni study a class of semilinear singularly perturbed equations using the method of fractional steps [8]. By the method of boundary layer function [9], Wang considered a kind of nonlinear singularly perturbed boundary value problems [10].

In this paper, we will discuss the interior layer for a class of nonlinear singularly perturbed differential-difference equations and construct its asymptotic expansion formula. Then, the existence of the smooth interior layer solution and the uniform validity of the asymptotic expansion are proved. The results of this paper are new and complement the previous ones.

We consider the following nonlinear singularly perturbed differential-difference equations

(1) μ2y′′=F(μy′(t),y(t),y(t−σ),t), t∈[0,2σ],y(t,μ)=α(t), −σ≤t≤0, y(2σ,μ)=y∗,

where

(2) $$\begin{array}{c} y\left(t\right)=\left(\begin{array}{c} y^{1}\left(t\right)\\ \vdots \\ y^{n}\left(t\right) \end{array}\right)\in R^{n},\;F=\left(\begin{array}{c} F^{1}\\ \vdots \\ F^{n} \end{array}\right)\in R^{n},\;0<\mu \ll 1, \end{array}$$

and functions *F* ^*i*^  (*i* = 1,…, *n*) are sufficiently smooth on the domain **D** = {(*μ* **y**′(*t*), **y**(*t*), *t*) | ||*μ* **y**′(*t*)|| ≤ *l*_1_, ||**y**(*t*)|| ≤ *l*_2_, 0 ≤ *t* ≤ 2*σ*}, and *l*_1_, *l*_2_ are given positive real numbers. The restriction on 2*σ* will not influence the essence of the problems.

We will use the method of fractional steps to discuss the system (1). Let *μ* = 0; then we can obtain the degenerate equations (3) and (4) of (1)

(3) $$\begin{array}{c} F\left(0,{\bar{y}}^{\left(1\right)}\left(t\right),\alpha \left(t-\sigma \right),t\right)=0,\;t\in \left[0,\sigma \right], \end{array}$$

(4) $$\begin{array}{c} F\left(0,{\bar{y}}^{\left(2\right)}\left(t\right),{\bar{y}}^{\left(1\right)}\left(t-\sigma \right),t\right)=0,\;t\in \left[0,2\sigma \right]. \end{array}$$

The degenerate problem (3) is solvable. We hypothesize that the solution of system (3) is ${\bar{y}}^{\left(1\right)}(t)=\varphi (t)$; substituting ${\bar{y}}^{\left(1\right)}(t)=\varphi (t)$ into (4) yields ${\bar{y}}^{\left(2\right)}(t)=\psi (t)$. Thus, we have the degenerate solution $\bar{y}(t)$ on the interval [0,2*σ*], namely, the following:

(5) $$\begin{array}{c} \bar{y}\left(t\right)=\{\begin{array}{cc} \varphi \left(t\right), & 0\leq t\leq \sigma ,\\ \psi \left(t\right), & \sigma \leq t\leq 2\sigma . \end{array} \end{array}$$

According to the truth of boundary layer functions and interior layer functions in [3], we can confirm that the interior layer may occur at *t* = *σ* and boundary layers may occur at the two terminal points of interval [0,2*σ*].

## 2. The Construction of Asymptotic Expansion in [0, *σ*]

In [0, *σ*], let *μ* **y**′ = **z**; the system (1) can be rewritten as

(6) μy′=z,μz′=F(μy′(t),y(t),α(t−σ),t),y(t,μ)=α(t), −σ≤t≤0,  y(2σ,μ)=y∗.

Let **x** ^(−)^ = (**y**, **z**)^*T*^ and using the method of boundary function [9], we can construct a series satisfying (6) in [0, *σ*]:

(7) $$\begin{array}{c} x^{\left(-\right)}=\bar{x}\left(t,\mu \right)+\Pi x\left(\tau_{0},\mu \right)+Q^{\left(-\right)}x\left(\tau ,\mu \right),\\ \tau_{0}=\frac{t}{\mu },\;\;\tau =\frac{t-\sigma }{\mu }, \end{array}$$

where

(8) $$\begin{array}{c} \bar{x}\left(t,\mu \right)={\bar{x}}_{0}\left(t\right)+\mu {\bar{x}}_{1}\left(t\right)+\cdots +\mu^{k}{\bar{x}}_{k}\left(t\right)+\cdots \end{array}$$

is called regular series of (6), while

(9) $$\begin{array}{c} \Pi x\left(\tau_{0},\mu \right)=\Pi_{0}x\left(\tau_{0}\right)+\mu \Pi_{1}x\left(\tau_{0}\right)+\cdots +\mu^{k}\Pi_{k}x\left(\tau_{0}\right)+\cdots \end{array}$$

is called the boundary series for *t* = 0, and

(10) Q(−)x(τ,μ)=Q0(−)x(τ)+μQ1(−)x(τ) +⋯+μkQk(−)x(τ)+⋯

is called the left boundary series for *t* = *σ*, and lim⁡_*τ*_0_→+*∞*_⁡Π_*k*_ **x**(*τ*_0_) = 0, lim⁡_*τ*→−*∞*_⁡*Q*_*k*_^(−)^ **x**(*τ*) = 0 hold. The system (6) has a continuous solution, so we assume that

(11) $$\begin{array}{c} y\left(\sigma ,\mu \right)=p_{0}+\mu p_{1}+\cdots +\mu^{k}p_{k}+\cdots , \end{array}$$

where **p**_*k*_(*k* = 0,1,…) are undetermined *n*-dimensional vector functions.

Put (7)–(10) into (6) and separate equations by measures *t*, *τ*_0_, *τ*; then we can write the regular part

(12) $$\begin{array}{c} {\bar{z}}_{0}\left(t\right)=0,\;\;F\left(0,\varphi \left(t\right),\alpha \left(t-\sigma \right),t\right)=0,\\ {\bar{y}}_{k-1}^{\prime }={\bar{z}}_{k}\left(t\right),\;\;{\bar{z}}_{k-1}^{\prime }\left(t\right)={\bar{F}}_{z}{\bar{z}}_{k}\left(t\right)+{\bar{F}}_{y}{\bar{y}}_{k}\left(t\right)+{\bar{h}}_{k}\left(t\right), \end{array}$$

where ${\bar{h}}_{k}(t)$ is a known vector function about ${\bar{z}}_{0}(t),{\bar{z}}_{1}(t),\ldots ,{\bar{z}}_{k-1}(t)$, ${\bar{y}}_{0}(t),{\bar{y}}_{1}\left(t\right),\ldots ,{\bar{y}}_{k-1}(t)$; elements of matrix ${\bar{F}}_{y}$, ${\bar{F}}_{z}$ take value at the point (0, *φ*(*t*), *α*(*t* − *σ*), *t*).

The first equation of (12) coincides with the left degradation problem (3), so we have ${\bar{y}}_{0}(t)=\varphi (t)$, ${\bar{z}}_{0}(t)=0$. To determine vector functions ${\bar{y}}_{k}(t)$, ${\bar{z}}_{k}(t)$, we need the following conditions.

(H1) Suppose that the determinant of ${\bar{F}}_{y}={\left(\partial F^{i}/\partial y^{i}\right)}_{n\times n}$ is not equal to zero at all times.

By (H1) and (12), ${\bar{y}}_{k}(t)$, ${\bar{z}}_{k}(t)$ can be completely determined.

(H2) Suppose that the characteristic equation of the systems (6) given by ${\left|\lambda^{2}I-{\bar{F}}_{z}\lambda -{\bar{F}}_{y}\right|}_{z=0,y=\varphi (t)}=0$ has 2*n* real valued solutions *λ*_*i*_(*t*), for *i* = 1,…, 2*n*, for all *t* ∈ [0, *σ*], where *Reλ*_*i*_(*t*) < 0, *i* = 1,…, *n*, *Reλ*_*i*_(*t*) > 0, *i* = *n* + 1,…, 2*n*, and **I** is the *n* × *n* identity matrix.

For Π_0_ *x*(*τ*_0_), we have

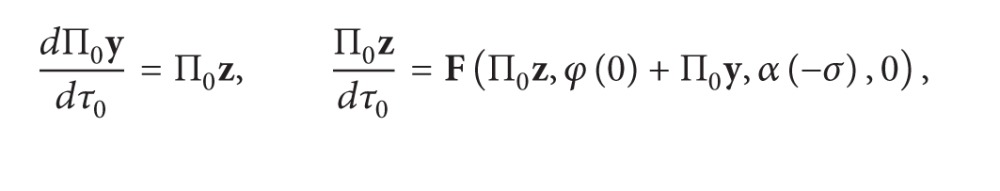
(13)

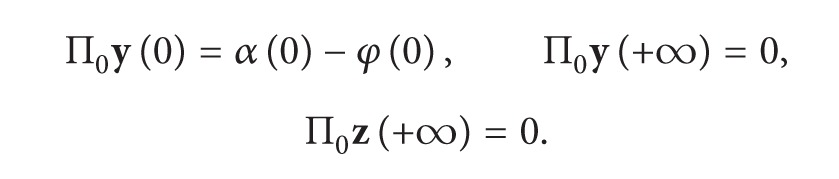
(14)
Let $\tilde{y}=\varphi (0)+\Pi_{0}y$, $\tilde{z}=\Pi_{0}z$; then the problems (13) and (14) can be changed into the following equations:

(15) dy~dτ=z~,  dz~dτ=F(z~,y~,α(−σ),0),y~(0)=α(0),  y~(−∞)=φ(0).

By (H2), there exists an *n*-dimensional stable manifold near the point (*φ*(0), 0) on the phase plane $\left(\tilde{y},\tilde{z}\right)$, which is in some region *G*_1_ of vector function Π_0_ **y**.

(H3) Suppose that the *n*-dimensional stable manifold is Π_0_ **z** = Φ^*L*^(Π_0_ **y**), and *α*(0) − *φ*(0) ∈ *G*_1_.

By (H2) and (H3), systems (13) and (14) have a solution Π_0_ **y**, Π_0_ **z**, which are both satisfied with exponential decay.

For Π_*k*_ **x**, we have

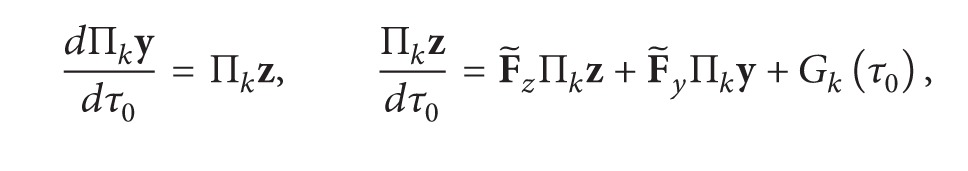
(16)

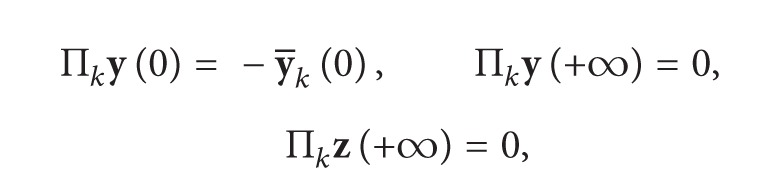
(17)
where ${\tilde{F}}_{z}$, ${\tilde{F}}_{y}$ take value at (Π_0_ **z**, *φ*(0) + Π_0_ **y**, *α*(−*σ*), 0) and *G*_*k*_(*τ*_0_) is a known vector function about Π_1_ **y**(*τ*_0_),…, Π_*k*−1_ **y**(*τ*_0_).

In fact, the homogeneous system of (16)

(18) $$\begin{array}{c} \frac{d\Pi_{k}y}{d\tau_{0}}=\Pi_{k}z,\;\;\frac{\Pi_{k}z}{d\tau_{0}}={\tilde{F}}_{z}\Pi_{k}z+{\tilde{F}}_{y}\Pi_{k}y \end{array}$$

is the variational equation of (13). Thus, it has a steady manifold

(19) $$\begin{array}{c} \Pi_{k}z=\frac{\partial \Phi^{L}\left(\Pi_{0}y\right)}{\partial y}\Pi_{k}y. \end{array}$$

Substituting (18) into (16), we have

(20) $$\begin{array}{c} \frac{\Pi_{k}y}{d\tau_{0}}=\frac{\partial \Phi^{L}}{\partial y}\Pi_{k}y. \end{array}$$

Let Π_*k*_ **y** = Φ_1_(*τ*_0_)*C* be the general solution of (20), under the boundary conditions (17), we obtain the general solution of (18) as

(21) (Πky(τ0))G=−Φ1(τ0)Φ1−1(0)y¯k(0),(Πkz(τ0))G=−∂ΦL(Π0y)∂yΦ1(τ0)Φ1−1(0)y¯k(0).

Next, set Π_k_*y, Π_k_*z be the particular solution of (16). Introducing a new transformation

(22) $$\begin{array}{c} \Pi_{k}^{*}y=\delta_{1},\;\;\Pi_{k}^{*}z=\frac{\partial \Phi^{L}\left(\Pi_{0}y\right)}{\partial y}\delta_{1}+\delta_{2} \end{array}$$

and substituting it into (16), we obtain the system

(23) $$\begin{array}{c} \frac{d\delta_{1}}{d\tau_{0}}=\frac{\partial \Phi^{L}\left(\Pi_{0}y\right)}{\partial y}\delta_{1}+\delta_{2},\\ \frac{d\delta_{2}}{d\tau_{0}}=\left({\tilde{F}}_{z}-\frac{\partial \Phi^{L}\left(\Pi_{0}y\right)}{\partial y}\right)\delta_{2}+G_{k}\left(\tau_{0}\right). \end{array}$$

Let *δ*_2_ = Ψ_1_(*τ*_0_)*C* be the general solution of $\left(d\delta_{2}/d\tau_{0}\right)=({\tilde{F}}_{z}-\left(\partial \Phi^{L}(\Pi_{0}y)/\partial y\right))\delta_{2}$; then we obtain a particular solution of (23) as

(24) $$\begin{array}{c} \delta_{2}\left(\tau_{0}\right)=\overset{\tau_{0}}{\underset{\infty }{\int }}\Psi_{1}\left(\tau_{0}\right)\Psi_{1}^{-1}\left(s\right)G_{k}\left(s\right)ds,\\ \delta_{1}\left(\tau_{0}\right)=\overset{\tau_{0}}{\underset{0}{\int }}\Phi_{1}\left(\tau_{0}\right)\Phi_{1}^{-1}\left(s\right)\overset{s}{\underset{\infty }{\int }}\Psi_{1}\left(s\right)\Psi_{1}^{-1}\left(t\right)G_{k}\left(t\right)dt\;ds. \end{array}$$

So a particular solution of (16) is given in the following form:

(25) Πk∗y(τ0) =∫0τ0Φ1(τ0)Φ1−1(s)∫∞sΨ1(s)Ψ1−1(t)Gk(t)dt ds,Πk∗z(τ0) =∂ΦL(Π0y)∂yΠk∗y(τ0)+∫∞τ0Ψ1(τ0)Ψ1−1(s)Gk(s)ds.

Hence, by virtue of (21) and (25), we obtain a solution of (16) and (17) as

(26) $$\begin{array}{c} \Pi_{k}y\left(\tau_{0}\right)=-\Phi_{1}\left(\tau_{0}\right)\Phi_{1}^{-1}\left(0\right){\bar{y}}_{k}\left(0\right)+\Pi_{k}^{*}y\left(\tau_{0}\right),\\ \Pi_{k}z\left(\tau_{0}\right)=-\frac{\partial \Phi^{L}\left(\Pi_{0}y\right)}{\partial y}\Phi_{1}\left(\tau_{0}\right)\Phi_{1}^{-1}\left(0\right){\bar{y}}_{k}\left(0\right)+\Pi_{k}^{*}z\left(\tau_{0}\right). \end{array}$$

Now, Π_*k*_ **x**(*τ*_0_) is completely determined. Obviously, Π_*k*_ **x**(*τ*_0_) decays exponentially as *τ* → *∞*.

**Lemma 1**

Under conditions (H1)–(H3), the boundary functions Π_*l*_ **x**(*τ*_0_) satisfy the following inequality:

(27) $$\begin{array}{c} ||\Pi_{l}x\left(\tau_{0}\right)||\leq C_{1}e^{-k_{1}\tau_{0}},\;\tau_{0}\geq 0,\;\;l=0,1,2,\ldots , \end{array}$$

where *C*_1_, *k*_1_ are all positive constants.

We first consider the system of *Q*_0_^(−)^ **x**

(28) $$\begin{array}{c} \frac{dQ_{0}^{\left(-\right)}y}{d\tau }=Q_{0}^{\left(-\right)}z,\\ \frac{dQ_{0}^{\left(-\right)}z}{d\tau }=F\left(Q_{0}^{\left(-\right)}z,\varphi \left(\sigma \right)+Q_{0}^{\left(-\right)}y,\alpha \left(0\right),\sigma \right),\\ Q_{0}^{\left(-\right)}y\left(0\right)=p_{0}-\varphi \left(\sigma \right),\\ Q_{0}^{\left(-\right)}y\left(-\infty \right)=Q_{0}^{\left(-\right)}z\left(-\infty \right)=0. \end{array}$$

Let $\varphi (\sigma )+Q_{0}^{\left(-\right)}y={\tilde{y}}_{l}$, $Q_{0}^{\left(-\right)}z={\tilde{z}}_{l}$, (28) can be written as

(29) dy~ldτ=z~l,  dz~ldτ=F(z~l,y~l,α(0),σ),y~l(0)=p(0),  y~l(−∞)=φ(σ).

By (H2), the equilibrium (*φ*(*σ*), 0) is a hyperbolical singular point on the plane $\left({\tilde{y}}_{l},{\tilde{z}}_{l}\right)$. There exists an *n*-dimensional stable manifold passing through (*φ*(*σ*), 0).

(H4) Suppose that this *n*-dimensional stable manifold is ${\tilde{z}}_{l}=\Phi^{\left(-\right)}({\tilde{y}}_{l})$, and ${\tilde{y}}_{l}(0)=p(0)\in G_{2}$, where *G*_2_ is a domain of ${\tilde{y}}_{l}$.

By (H4) and (28), *Q*_0_^(−)^ **x** can be completely determined, but it contains the unknown vector **p**_0_.

*Q*_*k*_^(−)^ **x** is determined by the following system:

(30) $$\begin{array}{c} \frac{dQ_{k}^{\left(-\right)}y}{d\tau }=Q_{k}^{\left(-\right)}z,\\ \frac{dQ_{k}^{\left(-\right)}z}{d\tau }={\tilde{F}}_{z}^{\left(-\right)}Q_{k}^{\left(-\right)}z+{\tilde{F}}_{y}^{\left(-\right)}Q_{k}^{\left(-\right)}y+H_{k}^{\left(-\right)}\left(\tau \right),\\ Q_{k}^{\left(-\right)}y\left(0\right)=p_{k}-{\bar{y}}_{k}\left(\sigma \right),\;\;Q_{k}^{\left(-\right)}y\left(-\infty \right)=Q_{k}^{\left(-\right)}z\left(-\infty \right)=0. \end{array}$$

Here *H*_*k*_^(−)^(*τ*) is a known vector; elements of matrix ${\tilde{F}}_{y}^{\left(-\right)}$, ${\tilde{F}}_{z}^{\left(-\right)}$ take value at the point (*Q*_0_^(−)^ **z**, *φ*(*σ*) + *Q*_0_^(−)^ **y**, *α*(0), *σ*). Because $\tilde{z}(\tau )=Q_{0}^{\prime }y$ is the solution of homogeneous systems of (30). By virtue of Liouville formula, we can obtain

(31) Qk(−)y=z~(τ)z~(0)[pk−y¯k(σ)] +z~(τ)∫0τdηz~2(η)h(η)∫−∞ηz~(s)h(s)Hk(−)(s)ds,

where $h(\tau )=\exp(-\int_{0}^{\tau_{0}}{\tilde{F}}_{z}d\tau )$. Thus, *Q*_*k*_^(−)^ **x**(*τ*) can be completely determined. We can easily obtain the exponential decay of *Q*_*k*_^(−)^ **x**(*τ*).

**Lemma 2**

Under the condition (H4), the boundary functions *Q*_*l*_ **x**(*τ*) satisfy the following inequality:

(32) $$\begin{array}{c} ||Q_{l}^{\left(-\right)}x\left(\tau \right)||\leq C_{2}e^{k_{2}\tau },\;\tau \leq 0,\;\;l=0,1,2,\ldots , \end{array}$$

where *C*_2_, *k*_2_ are positive constants.

## 3. The Construction of Asymptotic Expansion in [*σ*, 2*σ*]

In [*σ*, 2*σ*], let *μ* **y**′ = **z**; the system (1) can be rewritten as

(33) $$\begin{array}{c} \mu y^{\prime }=z,\\ \mu z^{\prime }=F\left(\mu y^{\prime }\left(t\right),y\left(t\right),\varphi \left(t-\sigma \right),t\right),\\ y\left(t,\mu \right)=\alpha \left(t\right),\;\;-\sigma \leq t\leq 0,\;\;y\left(2\sigma ,\mu \right)=y^{*}. \end{array}$$

Let **x** ^(+)^ = (**y**, **z**)^*T*^ and using the method of boundary function [9], we can construct a series formally satisfying (33) in [*σ*, 2*σ*]:

(34) x(+)=x¯¯(t,μ)+Q(+)x(τ,μ)+Rx(τ∗,μ),τ=t−σμ,  τ∗=t−2σμ,

where

(35) $$\begin{array}{c} \bar{\bar{x}}\left(t,\mu \right)={\bar{\bar{x}}}_{0}\left(t\right)+\mu {\bar{\bar{x}}}_{1}\left(t\right)+\cdots +\mu^{k}{\bar{\bar{x}}}_{k}\left(t\right)+\cdots \end{array}$$

is called regular series of (33), while

(36) Q(+)x(τ,μ)=Q0(+)x(τ)+μQ1(+)x(τ) +⋯+μkQk(+)x(τ)+⋯

is called the left boundary series for *t* = *σ*, and

(37) $$\begin{array}{c} Rx\left(\tau_{*},\mu \right)=R_{0}x\left(\tau_{*}\right)+\mu R_{1}x\left(\tau_{*}\right)+\cdots +\mu^{k}R_{k}x\left(\tau_{*}\right)+\cdots \end{array}$$

is called the boundary series for *t* = 2*σ*, and lim⁡_*τ*→+*∞*_⁡*Q*_*k*_^(+)^ **x**(*τ*) = 0, lim⁡_*τ*_∗_→−*∞*_⁡*R*_*k*_ **x**(*τ*_∗_) = 0 hold.

Substituting (35)–(37) into (33), separating *t*, *τ*, *τ*_∗_, and equating terms with same powers of *μ*, for $\bar{\bar{x}}$, we have

(38) z¯¯0(t)=0,  F(0,y¯¯0(t),φ(t−σ),t)=0,y¯¯0(t)=ψ(t),y¯¯k−1′=z¯¯k(t),  z¯¯k−1′(t)=F¯¯zz¯¯k(t)+F¯¯yy¯¯k(t)+h¯¯k(t),

where ${\bar{\bar{h}}}_{k}(t)$ is a known vector function about ${\bar{\bar{z}}}_{0}(t),{\bar{\bar{z}}}_{1}(t),\ldots ,{\bar{\bar{z}}}_{k-1}(t)$, ${\bar{\bar{y}}}_{0}(t),{\bar{\bar{y}}}_{1}(t),\ldots ,{\bar{\bar{y}}}_{k-1}(t)$; elements of matrix ${\bar{\bar{F}}}_{y}$, ${\bar{\bar{F}}}_{z}$ take value at the point (0, *ψ*(*t*), *φ*(*t* − *σ*), *t*). To determine the vector functions ${\bar{\bar{y}}}_{k}(t)$, ${\bar{\bar{z}}}_{k}(t)$, we need the following condition.

(H5) Suppose that the determinant of ${\bar{\bar{F}}}_{y}={\left(\partial F^{i}/\partial y^{i}\right)}_{n\times n}$ is not equal to zero at all times.

By (H5) and (38), ${\bar{\bar{y}}}_{k}(t)$, ${\bar{\bar{z}}}_{k}(t)$ can be completely determined.

For the zeroth approximation of the left boundary layer *Q*_0_^(+)^ **x**, we have

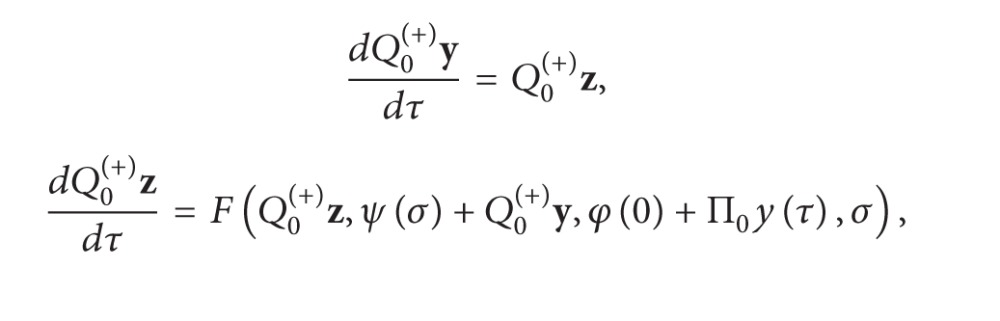
(39)

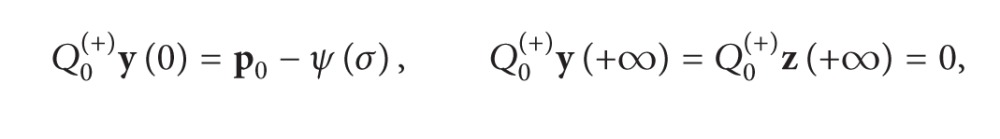
(40)
where *Q*_0_^(+)^ **x** is relevant to Π_0_ **y** and (39) is no longer an autonomous system. Combining with systems (13) and (14), we can discuss *Q*_0_^(+)^ **x**(*τ*). The combining equations and according conditions are

(41) dz~rdτ=F(z~r,y~r,y~,σ),  dy~rdτ=z~r,dz~dτ0=F(z~,y~,α(−σ),0),  dy~dτ0=z~,y~r(0)=p0,  y~r(+∞)=ψ(σ),y~(0)=α(0),  y~(+∞)=φ(0),

where the phase space $\left({\tilde{y}}_{r},{\tilde{z}}_{r},\tilde{y},\tilde{z}\right)$ is the direct sum of $\left({\tilde{y}}_{r},{\tilde{z}}_{r}\right)$ and $\left(\tilde{y},\tilde{z}\right)$.

(H6) Suppose that the characteristic equations of the systems (41) have 4*n* real valued solutions *λ*_*i*_(*t*), for *i* = 1,…, 4*n*, for all *t* ∈ [*σ*, 2*σ*], where *Reλ*_*i*_(*t*) < 0, *i* = 1,…, 2*n*, *Reλ*_*i*_(*t*) > 0, *i* = 2*n* + 1,…, 4*n*.

By condition (H6), there exists a 2*n*-dimensional stable manifold passing the equilibrium point of (*ψ*(*σ*), 0, *φ*(0), 0).

(H7) Suppose that this 2*n*-dimensional stable manifold is
  (42) $$\begin{array}{c} \vec{z}=\Phi^{\left(+\right)}\left(\tilde{y}\right), \end{array}$$

where $\vec{z}={\left(\Pi_{0}z,Q_{0}^{+}z\right)}^{T}$, $\vec{y}={\left(\Pi_{0}y,Q_{0}^{+}y\right)}^{T}$, Φ^(+)^ = (Φ_1_^(+)^, Φ_2_^(+)^)^*T*^, and (*α*(0) − *φ*(0), **p**_0_ − *ψ*(*σ*)) ∈ *G*_3_.

By (H7) and (39), (40), *Q*_0_^(+)^ **x**(*τ*) can be determined.

*Q*_*k*_^(+)^ **x**(*τ*) satisfies the following boundary value problem:

(43) $$\begin{array}{c} \frac{dQ_{k}^{\left(+\right)}y}{d\tau }=Q_{k}^{\left(+\right)}z,\\ \frac{dQ_{k}^{\left(+\right)}z}{d\tau }={\tilde{F}}_{z}^{\left(+\right)}Q_{k}^{\left(+\right)}z+{\tilde{F}}_{y}^{\left(+\right)}Q_{k}^{\left(+\right)}y+H_{k}^{\left(+\right)}\left(\tau \right),\\ Q_{k}^{\left(+\right)}y\left(0\right)=p_{k}-{\bar{\bar{y}}}_{k}\left(\sigma \right),\;\;Q_{k}^{\left(+\right)}y\left(+\infty \right)=Q_{k}^{\left(+\right)}z\left(+\infty \right)=0. \end{array}$$

By analogy with the left boundary terms, we have

(44) $$\begin{array}{c} Q_{k}^{\left(+\right)}y={\left(Q_{k}^{\left(+\right)}y\right)}^{G}+Q_{k}^{(+)*}y,\\ Q_{k}^{\left(+\right)}z={\left(Q_{k}^{\left(+\right)}z\right)}^{G}+Q_{k}^{(+)*}z, \end{array}$$

where

(45) (Qk(+)y)G=(pk−y¯¯k(σ))Φ2(τ0)Φ2−1(0),(Qk(+)z)G=∂Φ2(+)(Q0(+)y)∂yΦ2(τ)Φ2−1(0)(pk−y¯¯k(0)),Qk(+)∗y=∫0τΦ2(τ)Φ2−1(s)∫+∞sΨ2(s)Ψ2−1(t)Hk(+)(t)dt ds,Qk(+)∗z=∂Φ2(+)(Q0(+)y)∂yQk(+)∗y(τ)    +∫+∞τΨ2(τ)Ψ2−1(s)Hk(+)(s)ds.

The meaning of Φ_2_(*τ*) and Ψ_2_(*τ*) is similar to that of Φ and Ψ, respectively, but *Q*_*k*_^+^ **x**(*τ*) contains the unknown vector functions **p**_1_,…, **p**_*k*_.

**Lemma 3**

Under conditions (H5)–(H7), the boundary functions *Q*_*l*_^(+)^ **x**(*τ*) satisfy the following inequality:

(46) $$\begin{array}{c} ||Q_{l}^{\left(+\right)}x\left(\tau \right)||\leq C_{3}e^{-k_{3}\tau },\;\tau \geq 0, \end{array}$$

where *C*_3_, *k*_3_ are all positive constants.

For *R*_0_ *x*(*τ*_∗_), we have

(47) dR0ydτ∗=R0z,R0zdτ∗=F(R0z,ψ(2σ)+R0y,φ(σ),2σ),R0y(0)=y∗−ψ(2σ), R0y(−∞)=0, R0z(−∞)=0.

Consider the first approximate system of (47)

(48) (dR0ydτ∗dR0zdτ∗) =(0IF¯y(0,ψ(2σ),φ(σ),2σ)F¯z(0,ψ(2σ),φ(σ),2σ))  ×(R0yR0z).

In the same way, we can confirm that there exists an *n*-dimensional stable manifold, which is in some region *G*_4_ of vector function *R*_0_ **y**.

(H8) Suppose that the *n*-dimensional stable manifold is *R*_0_ **z** = Φ^*R*^(*R*_0_ **y**), and $y^{*}-{\bar{\bar{y}}}_{0}(2\sigma )\in G_{4}$.

By (H8), the system (47) has a solution. *R*_0_ **y** and *R*_0_ **z** are both satisfied with exponential decay estimate.

For *R*_*k*_ **x**, we have

(49) $$\begin{array}{c} \frac{dR_{k}y}{d\tau_{*}}=R_{k}z,\\ \frac{R_{k}z}{d\tau_{*}}={\tilde{\tilde{F}}}_{z}R_{k}z+{\tilde{\tilde{F}}}_{y}R_{k}y+G_{k}\left(\tau_{*}\right),\\ R_{k}y\left(0\right)=-{\bar{\bar{y}}}_{k}\left(2\sigma \right),\;R_{k}y\left(-\infty \right)=0,\;R_{k}z\left(-\infty \right)=0, \end{array}$$

where ${\tilde{F}}_{z}$, ${\tilde{F}}_{y}$ take value at $\left(R_{0}z,{\bar{\bar{y}}}_{0}(2\sigma )+R_{0}y,\varphi (\sigma ),2\sigma \right)$ and *G*_*k*_(*τ*_0_) is a known vector function.

The determination of *R*_*k*_ **x**(*τ*_∗_) is treated in the same way as Π_*k*_ **x**(*τ*_0_) and is omitted here. Obviously, *R*_*k*_ **x**(*τ*_∗_) satisfy the following Lemma.

**Lemma 4**

Under the condition (H8), the boundary functions *R*_*l*_ **x**(*τ*_∗_) satisfy the following inequality:

(50) $$\begin{array}{c} ||R_{l}x\left(\tau_{*}\right)||\leq C_{4}e^{k_{4}\tau_{*}},\;\tau_{*}\leq 0, \end{array}$$

where *C*_4_, *k*_4_ are all positive constants.

Obviously, *Q*_*k*_^(−)^ **x**(*τ*), *Q*_*k*_^(+)^ **x**(*τ*) contain the unknown vector functions **p**_0_,…, **p**_*k*_. Next, we will use the continuous conditions to determine them.

As the solution of the original problem is continuous at *t* = *σ*, the solution *x*(*t*, *μ*) of equivalent system needs to satisfy

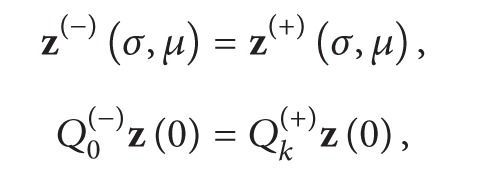
(51)

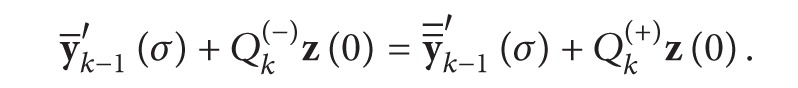
(52)
First, we will seek the value of **p**_0_. (H4), (H7), and (51) show

(53) $$\begin{array}{c} \Phi^{l}\left(p_{0}-\varphi \left(\sigma \right),0\right)=\Phi_{2}^{\left(+\right)}\left(p_{0}-\psi \left(\sigma \right),0\right). \end{array}$$

Let

(54) $$\begin{array}{c} N_{0}\left(p_{0}\right)=\Phi^{l}\left(p_{0}-\varphi \left(\sigma \right),0\right)-\Phi_{2}^{\left(+\right)}\left(p_{0}-\psi \left(\sigma \right),0\right)=0. \end{array}$$

(H9) Suppose that (54) has a solution $p_{0}={\bar{p}}_{0}$ and ${\left(\partial N_{0}/\partial p_{0}\right)|}_{y={\bar{p}}_{0}}\neq 0$.

Next, bringing (30) and (39) into (52), we have

(55) Nk(pk)=y¯k−1′(σ)+Qk(−)z(0)−y¯¯k′(σ)−Qk(+)z(0)=0,Qk(−)z(0)=z~′(0)z~(0)[pk−y¯k(σ)]+1z~(0)∫−∞0z~(s)h(s)Hk(−)ds,Qk(+)z(0)=∂Φ2(+)(Q0(+)y)∂y[pk−y¯¯k(0)] +∫+∞0Ψ2(τ)Ψ2−1(s)Hk(+)(s)ds.

By (55), **p**_*k*_ can be completely determined.

## 4. The Main Result

Let

(56) $$\begin{array}{c} X_{n}\left(t,\mu \right)=\{\begin{array}{cc} \overset{n}{\underset{i=0}{\sum }}\mu^{i}{\bar{x}}_{i}\left(t\right)+\Pi_{i}x\left(\tau_{0}\right)+Q_{i}^{\left(-\right)}x\left(\tau \right), & 0\leq t\leq \sigma ,\\ \overset{n}{\underset{i=0}{\sum }}\mu^{i}{\bar{x}}_{i}\left(t\right)+Q_{i}^{\left(+\right)}x\left(\tau \right)+R_{i}x\left(\tau_{*}\right), & \sigma \leq t\leq 2\sigma . \end{array} \end{array}$$

**Theorem 5**

Under conditions (H1)–(H9) and Lemma 1–Lemma 4, there exist positive constants *μ*_0_ > 0, *c* > 0, such that, for 0 < *μ* ≤ *μ*_0_, the solution **x**(*t*, *μ*) of the systems (1) exists in the interval [0,2*σ*] and satisfies the inequality

(57) $$\begin{array}{c} ||x\left(t,\mu \right)-X_{n}\left(t,\mu \right)||\leq c\mu^{n+1}. \end{array}$$

## 5. Example

Let us consider the systems

(58) μ2y′′=y(t)−y(t−12),y(t)=t, t∈[−12,0], y(1)=12,

where **y** = (*y*_1_, *y*_2_)^*T*^. For 0 ≤ *t* ≤ 1, the degenerate solution of (58) is

(59) $$\begin{array}{c} \varphi \left(t\right)={\left(t-\frac{1}{2},t-\frac{1}{2}\right)}^{T},\;0\leq t\leq \frac{1}{2},\\ \psi \left(t\right)={\left(t-1,t-1\right)}^{T},\;\frac{1}{2}\leq t\leq 1. \end{array}$$

Obviously, conditions (H2) and (H3) hold. Π_0_ **y**(*τ*_0_), *R*_0_ **y**(*τ*_∗_), *Q*_0_^(−)^ **y**(*τ*), and *Q*_0_^(+)^ **y**(*τ*) are given by the following systems, respectively,

(60) d2Π0ydτ02=Π0y,  Π0y(0)=(12,12)T,Π0y(+∞)=0,d2R0ydτ∗2=R0y,  R0y(0)=(12,12)T,R0y(−∞)=0,d2Q0(−)ydτ2=Q0(−)y,  Q0(−)y(0)=(p01,p02)T,Q0(−)y(−∞)=0,d2Q0(+)ydτ2=Q0(+)y,  Q0(+)y(0)=(p01+12,p02+12)T,Q0(+)y(+∞)=0.

After simple manipulations, we obtain Π_0_ **y** = ((1/2)*e* ^−*τ*_0_^, (1/2)*e* ^−*τ*_0_^)^*T*^, *R*_0_ **y** = ((1/2)*e* ^*τ*_∗_^, (1/2)*e* ^*τ*_∗_^)^*T*^, *Q*_0_^(−)^ **y**(*τ*) = (*p*_01_ *e* ^*τ*^, *p*_02_ *e* ^*τ*^)^*T*^, *Q*_0_^(+)^ **y**(*τ*) = (*p*_01_ *e* ^−*τ*^ + (1/2)*e* ^−*τ*^ + (1/4)*τe* ^−*τ*^, *p*_02_ *e* ^−*τ*^ + (1/2)*e* ^−*τ*^ + (1/4)*τe* ^−*τ*^)^*T*^. By the smooth connection (*dQ*_0_^(−)^ **y**/*dτ*)|_*τ*=0_ = (*dQ*_0_^(+)^ **y**/*dτ*)|_*τ*=0_, we have *H*(**p**_0_) = (2*p*_01_ + (1/2) − (1/4), 2*p*_02_ + (1/2) − (1/4))^*T*^ = 0. Obviously, **p**_0_ = (−1/8, −1/8)^*T*^ and |*H*(**p**_0_)/*d* **p**_0_|_**p**_0_=−1/8_ = 4 ≠ 0. Thus, we obtain that the zero order asymptotic solution of (58) is

(61) y(t,μ) =(t−12+12e−t/μ−18e(2t−1)/2μ,    t−12+12e−t/μ−18e(2t−1)/2μ)T, 0≤t≤12,y(t,μ) =(t−1+38e(−2t−1)/2μ+2t−18μe(−2t−1)/2μ+12e(t−1)/μ,    t−1+38e(−2t−1)/2μ+2t−18μe(−2t−1)/2μ+12e(t−1)/μ)T,12≤t≤1.

We can see that the zero order asymptotic solution is close to the reduced solution.

## 6. Conclusive Remarks

Using the boundary layer function method, we consider a class of *n*-dimensional singularly perturbed differential equations with time delay. Under some assumptions, we obtain the asymptotic solution of the system (1). In comparison with [7] an [9], the system we study is more general. We use functional method to solve the asymptotic solution of (1) in this paper. It is different from the numerical solution obtained by numerical method. The asymptotic solution of (1) can be used in analytic calculation and obtain the asymptotic behaviors for deeper physical quantities.
